# Sensing of Surface and Bulk Refractive Index Using Magnetophotonic Crystal with Hybrid Magneto-Optical Response

**DOI:** 10.3390/s21061984

**Published:** 2021-03-11

**Authors:** Daria Ignatyeva, Pavel Kapralov, Polina Golovko, Polina Shilina, Anastasiya Khramova, Sergey Sekatskii, Mohammad Nur-E-Alam, Kamal Alameh, Mikhail Vasiliev, Andrey Kalish, Vladimir Belotelov

**Affiliations:** 1 Russian Quantum Center, 121205 Moscow, Russia; ignatyeva@physics.msu.ru (D.I.); kapralov_pavel@mail.ru (P.K.); ae.khramova@gmail.com (A.K.); kalish@physics.msu.ru (A.K.); 2 Faculty of Physics, Lomonosov Moscow State University, 119991 Moscow, Russia; golovko.pv18@physics.msu.ru; 3 Institute of Physics and Technology, V.I. Vernadsky Crimean Federal University, 295007 Simferopol, Russia; 4 Faculty of Physics, National Research University Higher School of Economics, 101000 Moscow, Russia; polly.penkina@gmail.com; 5 Center for Photonics and 2D Materials, Moscow Institute of Physics and Technology, 117303 Dolgoprudny, Russia; 6 Laboratory of Biological Electron Microscopy, Institute of the Physics of Ecole Polytechnique Fédérale de Lausanne, 1015 Lausanne, Switzerland; serguei.sekatski@epfl.ch; 7 Electron Science Research Institute, Edith Cowan University, Perth, WA 6027, Australia; m.nur-e-alam@ecu.edu.au (M.N.-E.-A.); k.alameh@ecu.edu.au (K.A.); vasiliev.mikhail@gmail.com (M.V.)

**Keywords:** optical sensor, photonic crystal, surface optical wave, magneto-optics, transverse magneto-optical Kerr effect, Faraday effect

## Abstract

We propose an all-dielectric magneto-photonic crystal with a hybrid magneto-optical response that allows for the simultaneous measurements of the surface and bulk refractive index of the analyzed substance. The approach is based on two different spectral features of the magneto-optical response corresponding to the resonances in p- and s-polarizations of the incident light. Angular spectra of p-polarized light have a step-like behavior near the total internal reflection angle which position is sensitive to the bulk refractive index. S-polarized light excites the TE-polarized optical Tamm surface mode localized in a submicron region near the photonic crystal surface and is sensitive to the refractive index of the near-surface analyte. We propose to measure a hybrid magneto-optical intensity modulation of p-polarized light obtained by switching the magnetic field between the transverse and polar configurations. The transversal component of the external magnetic field is responsible for the magneto-optical resonance near total internal reflection conditions, and the polar component reveals the resonance of the Tamm surface mode. Therefore, both surface- and bulk-associated features are present in the magneto-optical spectra of the p-polarized light.

## 1. Introduction

Optical sensors based on the surface waves are characterized by a very high sensitivity that is crucial for various practical applications, including biosensing, medical studies, detection of chemical species, food safety, and environmental control [1]. Most commercially available sensors are based on the detection of the surface plasmon resonance that shifts under the variation of the refractive index of the analyte, which is an analyzed substance in the sensor measurement cell. This technique is very convenient in the sense of the simplicity of the optical measurements and the structures required for sensing. Although it can be scaled down to construct nanosensors utilizing plasmonic nanoantennas [2,3], in the present study we focus on the planar structures that allow operating with the macroscopic measurement cells and utilize the flat sensing surfaces to attach and detect various biological species [4,5]. Alongside with these advantages, surface plasmon resonances are characterized by low quality factors *Q*~10 due to the high absorption in the metallic layers which limit the sensitivity of such sensors.

Many recent studies are focused on the different approaches to increase the Q-factors of the surface wave resonances and to overcome this limitation. For example, long-range propagating surface plasmon polaritons in thin metal films deposited on the top of a dielectric layer [6,7,8], multilayered plasmonic [9,10] structures or photonic crystals [11,12,13,14,15] produce narrow optical resonances and allow for the Q-factor increase. Even more narrow resonances could be produced using all-dielectric structures [16] instead of plasmonic ones, and utilization of photonic crystals with Tamm surface optical modes. Actually, this approach allows not only to increase the Q-factor, but also to achieve the sensitivity to the bulk and surface refractive indices due to the measurements performed simultaneously in p- and s-polarizations [17,18]. This possibility is not available in any kind of the plasmonic structures. Another possibility to increase the resonance Q-factor is to introduce a magnetic material in the sensing structure and to utilize the magneto-optical measurements of the transverse magneto-optical Kerr effect (TMOKE) instead of optical reflectance spectra [5,19,20,21,22]. The magneto-optical response can be enhanced with a variety of metamaterials [23,24,25,26], plasmonic coatings, dielectric nanostructures [27,28,29], and photonic crystals [30,31,32,33].

A combination of these two approaches, excitation of a photonic-crystal supported Tamm surface mode and performing the magneto-optical measurements, was recently reported to be very promising from the point of view of dramatical increase of the sensor sensitivity [30,31,32]. However, as the TMOKE is observed only in the p-polarization of the incident light, this approach does not allow to perform magneto-optical measurements in a dual-channel regime for a simultaneous detection of the bulk and surface refractive indices of the analyte. Similar increase of the sensitivity was predicted for the polarization-based measurements in the polar configuration of a magnetic field for a case when the resonances in p- and s-polarizations coincide [33]. However, it also does not provide any possibility for the dual-channel measurements.

In the present study, we focus on an all-dielectric magnetophotonic crystal (MPC)-based structure with a hybrid magneto-optical response that allows us to overcome this limitation. Optical response of the designed structure has two different spectral features in p- and s-polarizations of the incident light similar to the previously reported nonmagnetic structures [17,18]. Angular spectra of p-polarized light have a step-like behavior near the total internal reflection angle which position depends on the bulk refractive index. S-polarized light excites the TE-polarized Tamm surface mode localized in a submicron region near the photonic crystal surface that is sensitive to the refractive index of this near-surface analyte. Instead of the conventionally measured TMOKE, we propose to measure a magneto-optical intensity modulation of p-polarized light obtained by switching the magnetic field between the transverse and polar configurations. The transversal component of the external magnetic field is responsible for the appearance of the spectral feature associated with a total internal reflection, and the polar component leads to the polarization mixing and appearance of the magneto-optical resonance corresponding to the Tamm surface mode. Therefore, both surface- and bulk-associated features are present in magneto-optical spectra of a selected light polarization.

## 2. Materials and Methods

**Sample design and numerical simulations**. An impedance method described in [34] was used to design the magneto-photonic crystal supporting the TE-polarized Tamm surface mode at the interface between the magnetophotonic crystal and the water as analyte. A thin-film based semitransparent magneto-optical material (bismuth-substituted iron-garnet, BIG) layer was selected as a terminating magnetic layer of the photonic crystal. The parameters of the magnetophotonic crystal (MPC) were chosen to provide the surface mode at the wavelength of 771 nm which corresponds to the operating wavelength of the laser diode. MPC was designed as follows: two pairs of the Ta_2_O_5_/SiO_2_ layers with the thicknesses of 143 nm/243 nm, correspondingly, covered by a terminating 220-nm thick bismuth-substituted iron-garnet film.

We used self-made software based on transfer matrix technique, the details of which are presented, for example, in [35]. It allows calculation of intensity and polarization of reflected and transmitted light for layered structures.

**Sample fabrication**. The magnetophotonic crystal was fabricated in a two-stage process. First, a photonic crystal containing Ta_2_O_5_/SiO_2_ layers with the designed parameters was deposited onto a SiO_2_ substrate by magnetron sputtering. Secondly, a bismuth-substituted iron garnet compound doped with dysprosium and gallium layer was prepared on top of the PC using the RF magnetron sputtering technique. The sputtering targets used had nominal compositions of Bi_2.1_Dy_0.9_Fe_3.9_Ga_1.1_O_12_. This type of garnet layer possessed simultaneously a high Faraday rotation and the necessary level of uniaxial magnetic anisotropy to orient the magnetization of the films in the direction perpendicular to the film plane. The sputter deposition process parameters used to prepare the garnet layer on top of PCs are detailed in Table 1.

After sputtering the sample was annealed at 600 °C to provide ferromagnetic state of the garnet layer.

**Figure 1:**
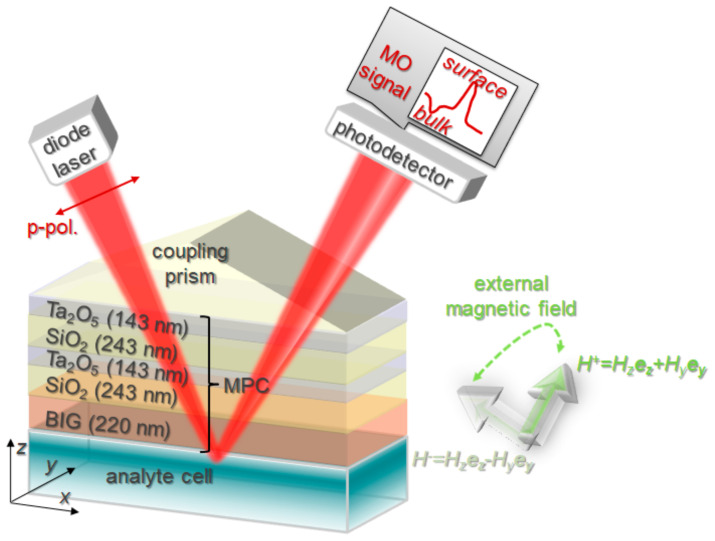
Principle scheme of the magneto-optical measurements.

**Experimental setup**. MPC was attached to a coupling SiO_2_ prism with the base angle of 60°. It was irradiated by a laser diode at a wavelength of 771 nm. Measurements were held for s- and p-polarization of the laser beam separately. The laser beam was modulated with an optical chopper operating at a frequency of 360 Hz for measurements of the reflection. MPC with the prism were mounted on the rotating platform enabling to vary the angle of light incidence with accuracy of 0.05°. The reflected light was detected by a photodiode. An external magnetic field was applied. It had two components—the in-plane component alternating in time with an amplitude of 120 mT and the out-of-plane component of a constant value of 40 mT (along *z*-axis, see Figure 1). Therefore, the external magnetic field was switching between two directions—H^+^ and H^−^ in YZ-plane. H^+^ and H^−^ are directed at angles of 70° and −70° to *z*-axis, respectively.

**Figure 2:**
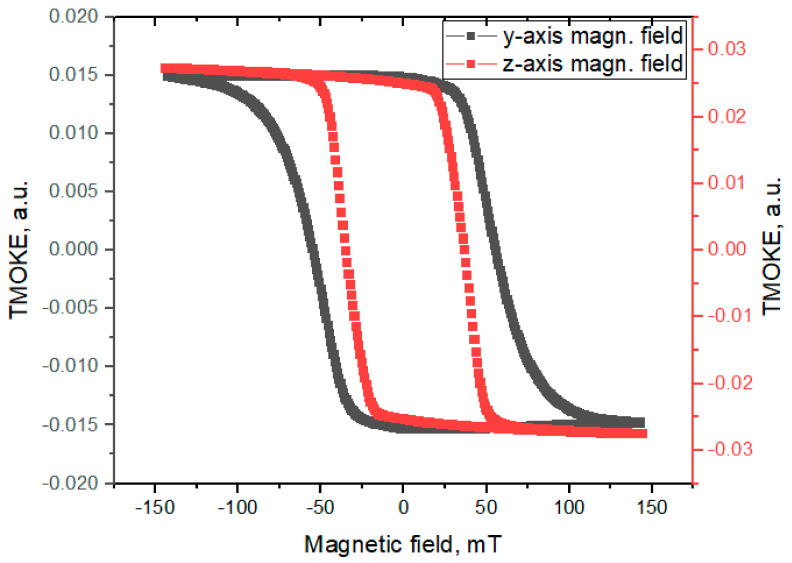
The hysteresis for magnetic field applied along the *y*-axis and along the *z*-axis.

The hysteresis obtained in external magnetic field directed along the *z*-axis and along the *y*-axis are shown in Figure 2.

The measured magneto-optical effect is defined as magneto-optical modulation of the optical reflectance:

(1) $$\delta =\frac{R\left(H^{+}\right)-R\left(H^{-}\right)}{R\left(H^{+}\right)+R\left(H^{-}\right)}.$$

## 3. Results

The measurement scheme that was used for the magneto-optical refractive index sensing exploiting the MPC with a hybrid magneto-optical response is presented in Figure 1.

**Figure 3:**
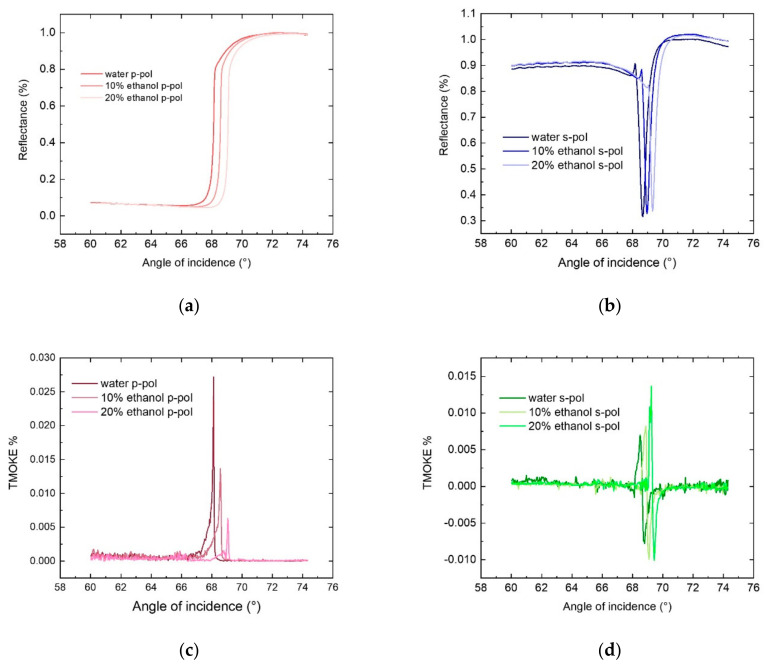
The reflectance (**a**,**b**) and magneto-optical (**c**,**d**) angular spectra of the magnetophotonic crystal in (**a**,**c**) p- and (**b**,**d**) s-polarizations of the incident light measured for different concentrations of the ethanol.

All-dielectric magneto-photonic crystal with a bismuth-substituted iron-garnet film exhibits a step-like behavior of the reflectance coefficient near the total internal reflection angle in p-polarization of the incident light, as shown in Figure 3a. The position of this spectral feature is determined by the total internal reflection angle $\theta_{TIR}=\sin^{-1}\left(n_{an}/n_{prism}\right)$ between the prism material and the analyzed substance with $n_{an}$ refractive index. Reflectance spectra of the p-polarized light is sensitive to the external magnetic field applied in the transverse configuration (namely, switching between the $+H_{y}$ and $-H_{y}$ component). This causes the magneto-optical spectra to have a resonance near $\theta_{TIR}$ (Figure 3c) which position is predominantly associated with a bulk refractive index of the analyte.

On the other hand, the MPC is designed to support a TE-polarized Tamm surface mode that manifests itself as a dip in the reflectance spectra of the s-polarized incident light (Figure 3b). This mode itself is completely insensitive to $H_{y}$ component of the magnetic field [32]. However, if a magnetic field in polar configuration ($H_{z}$) is applied, the eigen polarization of the mode changes [36,37] and it acquires TM-components that were absent in the nonmagnetic case. In its turn, the emerged TM-components are sensitive to the switching of the direction of the $H_{y}$ component of the external magnetic field. Thus, this mixing of the TE- and TM-components of the electromagnetic field due to the eigen polarization change is observed as a resonance in the magneto-optical spectra of the s-polarized light, see Figure 3d.

Therefore, in contrast to the various configurations of the magneto-optical sensors studied before, the proposed configuration allows one to measure the magneto-optical response in the dual-channel regime, in p- and s-polarizations independently. As it will be shown further, these two channels are responsible for the measurement of the bulk and surface refractive indices of the analyte. At the same time the proposed scheme preserves the advantage of the magneto-optical sensing which is an extremely high figure of merit.

Figure 3 shows the results of the performed sensing experiments where both optical and magneto-optical response were measured for different concentrations of the ethanol in water solution (mixtures are made in volume). Taking the refractive indices of water $n_{wat}=1.3330$ [38] and ethanol $n_{et}=1.3611$ [39] at the room temperature, and assuming the linear dependence of the refractive index on the ethanol concentration, one may obtain $n_{10\%etanol}=1.33581$, $n_{20\%etanol}=1.33862$. This gives the sensitivity of the position of the magneto-optical resonances $S=\partial \theta_{res}/\partial n$ equal to $S_{p}=168\;\deg./\mathrm{RIU}$, $S_{s}=130\;\deg./\mathrm{RIU}$. Figure of merits for these configurations determined as the ratio of the sensitivity to the full-width at half-maximum of the resonances, FOM=S/Δθ, is estimated as $FOM_{p}=982\;{\mathrm{RIU}}^{-1}$ for p-polarized light, and $FOM_{s}=521\;{\mathrm{RIU}}^{-1}$ for s-polarized light, which is about an order higher than typical values for the plasmonic sensors. In the case of plasmonic sensors, FOM is limited by material losses in the metal and reaches values of ≈20 ${\mathrm{RIU}}^{-1}$ [40,41]. For dielectric nanostructures FOM is limited by material losses in the dielectric and reaches values of ≈300 ${\mathrm{RIU}}^{-1}$ [42,43].

## 4. Discussion

**Figure 4:**
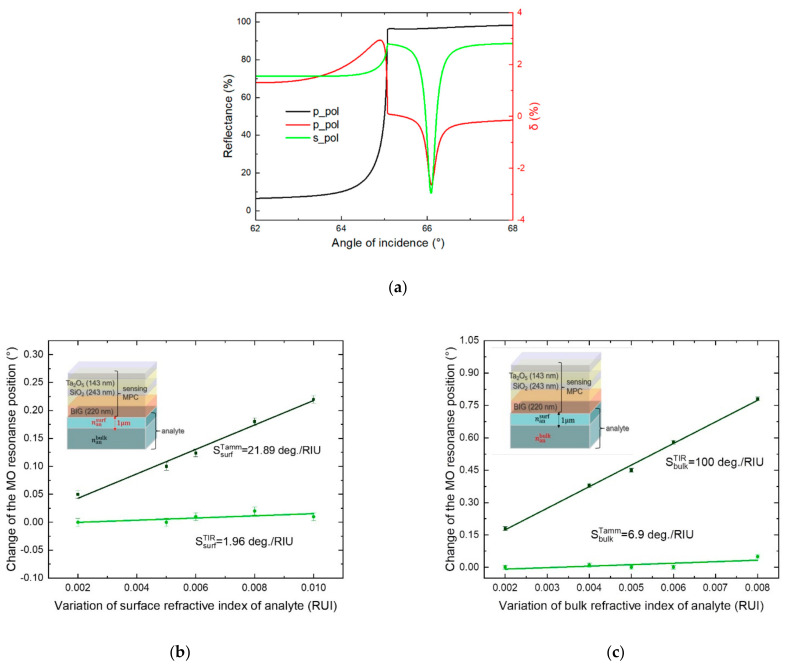
Surface and bulk refractive index sensing. (**a**) Reflectance angular spectra of the p- and s-polarized light; magneto-optical modulation R_p_(H_z_)-R_p_(H_y_). (**b**,**c**) Sensitivity of the two magneto-optical resonances to the variation of the near-surface (**b**) and bulk (**c**) analyte refractive index.

Let us now discuss the dual-channel magneto-optical measurements in more details to reveal other advantages of this approach. The experimental measurements were performed using p- and s-polarized incident light in order to get the two resonances of the magneto-optical effect: one associated with the total internal reflection (TIR), and the other one associated with the Tamm surface mode. Actually, if the magnetic field direction were changed and instead of tilting of **H**-vector with respect to the *z*-axis one will switch between $H_{z}$ and $H_{y}$ directions of the magnetic field, both magneto-optical resonances would be observed in the angle resolved reflectance spectra of the p-polarized light, as shown in Figure 4a. The resonance of the TE-polarized Tamm surface mode would be excited by p-polarized light due to the coupling between the TM and TE components if the $H_{z}$ magnetic field is applied, and would not reveal itself if $H_{y}$ magnetic field is applied. This would lead to the magneto-optical modulation of the light reflected from the interface.

An important point is a significantly different penetration depth of the electromagnetic field in the analyte for the TIR and Tamm-associated magneto-optical resonances. Actually, the magneto-optical resonance in p-polarization corresponds to the total internal reflection angle where the penetration depth in the analyte is very high (it equals infinity exactly at the total internal reflection angle). The energy of the Tamm mode is localized in a submicron region $L_{TE}$ near the interface of BIG and analyte:

(2) $$L_{TE}=\frac{\lambda }{4\pi }\frac{1}{\sqrt{n_{pr}^{2}sin^{2}\theta -n_{an}^{2}}},$$

and for the considered structure $L_{TE}=0.38\;\mu m$. Therefore, the two magneto-optical resonances corresponding to TIR and Tamm mode excitation which are observed for the slightly different angles have a drastically different penetration depth in the analyzed substance. This is a key to the sensing of the bulk and refractive indices simultaneously in a frame of a single device and single-polarization measurements.

To show this, we performed additional numerical simulations of the magneto-optical response of the two-component analyte with varying surface or bulk refractive indices. The analyte layer with so-called surface refractive index $n_{an}^{surf}$ having a thickness of 1 μm is located near the magnetophotonic crystal surface and mimics the measured substance attached to the surface of the sensor. The thickness of this layer was selected to match, on the one hand, the penetration depth of the electromagnetic field (which equals to $2L_{TE}$), and, on the other hand, typical size of different biological objects, such as bacteria, cells, etc. Actually, the sensor is suitable for measurements of various biological objects with the characteristic sizes ranging from 0.2 to 1.5 μm approximately, which include various types of bacteria, such as nanobacteria and ultramicrobacteria (having the size from 200 to 800 nanometers); prokaryotic cells (in most cases having the size from 0.5 to 3 µm), microplasma cells and many others. The semi-infinite layer of $n_{an}^{bulk}$ corresponds to the whole analyte solution in the measurement cell, which refractive index generally may change due to some temperature fluctuations, changes of the composition of the solution, etc.

Figure 4b,c shows that, according to the qualitative considerations provided above, the TIR-associated magneto-optical resonance is sensitive mainly to the bulk refractive index, while the Tamm-associated resonance depends mostly on the surface one. The numerically obtained sensitivities are 100 deg./RIU and 21.89 deg./RIU for the corresponding magneto-optical resonance positions. Therefore, the proposed approach makes it possible to measure simultaneously and with a high sensitivity the surface and bulk refractive indices of analyte using a single-polarization magneto-optical intensity effect angular spectra.

Therefore, we showed both experimentally and numerically that all-dielectric magneto-photonic crystal with a hybrid magneto-optical response allows for the simultaneous measurements of the surface and bulk refractive indexes of the analyzed substance. The measurements were performed using liquids as analytes, therefore, the demonstrated sensor with a hybrid magneto-optical response is ready for operation with real biological samples.

## Figures and Tables

**Table 1:** Summary of process parameters used to prepare the garnet layers.

Process Parameters	Values and Comments
Sputtering target stoichiometry oxide-mixed garnet targets	Bi_2.1_Dy_0.9_Fe_3.9_Ga_1.1_O_12_
Base pressure	4–5 × 10^−6^ Torr
Argon (Ar) pressure	≈2 mTorr
Substrate stage temperature	Room temperature 21–23 °C
Substrate stage rotation rate	16–17 rpm

## Data Availability

The data presented in this study are contained within the article.

## References

[1] Homola J. (2008). Surface plasmon resonance sensors for detection of chemical and biological species. Chem. Rev..

[2] Herreño-Fierro C.A., Patiño E.J., Armelles G., Cebollada A. (2016). Surface sensitivity of optical and magneto-optical and ellipsometric properties in magnetoplasmonic nanodisks. Appl. Phys. Lett..

[3] Maccaferri N., Gregorczyk K.E., de Oliveira T.A.G., Kataja M., van Dijken S., Pirzadeh Z., Dmitriev A., Åkerman J., Knez M., Vavassori P. (2015). Ultrasensitive and label-free molecular-level detection enabled by light phase control in magnetoplasmonic nanoantennas. Nat. Commun..

[4] Rostova E., Adiba C.B., Dietler G., Sekatskii S. (2016). Kinetics of Antibody Binding to Membranes of Living Bacteria Measured by a Photonic Crystal-Based Biosensor. Biosensors.

[5] Manera M.G., Ferreiro-Vila E., Garcia-Martin J.M., Garcia-Martin A., Rella R. (2014). Enhanced antibody recognition with a magneto-optic surface plasmon resonance (MO-SPR) sensor. Biosens. Bioelectron..

[6] Slavík R., Homola J. (2007). Ultrahigh resolution long range surface plasmon-based sensor. Sens. Actuators B.

[7] Ruan B., You Q., Zhu J., Wu L., Guo J., Dai X., Xiang Y. (2018). Improving the performance of an SPR biosensor using long-range surface plasmon of Ga-doped zinc oxide. Sensors.

[8] Wark A.W., Lee H.J., Corn R.M. (2005). Long-range surface plasmon resonance imaging for bioaffinity sensors. Anal. Chem..

[9] Qin J., Zhang Y., Liang X., Liu C., Wang C., Kang T., Lu H., Zhang L.S., Zhou P., Wang X. (2017). Ultrahigh figure-of-merit in metal-insulator-metal magnetoplasmonic sensors using low loss magneto-optical oxide thin films. ACS Photon..

[10] Floess D., Chin J.Y., Kawatani A., Dregely D., Habermeier H.U., Weiss T., Giessen H. (2015). Tunable and switchable polarization rotation with non-reciprocal plasmonic thin films at designated wavelengths. Light Sci. Appl..

[11] Sasin M.E., Seisyan R.P., Kalitteevski M.A., Brand S., Abram R.A., Chamberlain J.M., Egorov A.Y., Vasil’ev A.P., Mikhrin V.S., Kavokin A.V. (2008). Tamm plasmon polaritons: Slow and spatially compact light. Appl. Phys. Lett..

[12] Pankin P.S., Vetrov S.Y., Timofeev I.V. (2017). Tunable hybrid Tamm-microcavity states. J. Opt. Soc. Am. B.

[13] Konopsky V.N., Alieva E.V. (2006). Long-range propagation of plasmon polaritons in a thin metal film on a one-dimensional photonic crystal surface. Phys. Rev. Lett..

[14] Konopsky V.N., Alieva E.V. (2009). Long-range plasmons in lossy metal films on photonic crystal surfaces. Opt. Lett..

[15] Sekatskii S.K., Smirnov S., Dietler G., Alam M.N., Vasiliev M., Alameh K. (2018). Photonic crystal-supported long-range surface plasmon-polaritons propagating along high-quality silver nanofilms. Appl. Sci..

[16] Christofi A., Kawaguchi Y., Alù A., Khanikaev A.B. (2018). Giant enhancement of Faraday rotation due to electromagnetically induced transparency in all-dielectric magneto-optical metasurfaces. Opt. Lett..

[17] Konopsky V.N., Karakouz T., Alieva E.V., Vicario C., Sekatskii S.K., Dietler G. (2013). Photonic crystal biosensor based on optical surface waves. Sensors.

[18] Konopsky V.N., Alieva E.V. (2007). Photonic crystal surface waves for optical biosensors. Anal. Chem..

[19] Martín-Becerra D., Armelles G., González M.U., García-Martín A. (2013). Plasmonic and magnetoplasmonic interferometry for sensing. N. J. Phys..

[20] Armelles G., Cebollada A., García-Martín A., González M.U. (2013). Magnetoplasmonics: Combining magnetic and plasmonic functionalities. Adv. Opt. Mat..

[21] Regatos D., Fariña D., Calle A., Cebollada A., Sepúlveda B., Armelles G., Lechuga L.M. (2010). Au/Fe/Au multilayer transducers for magneto-optic surface plasmon resonance sensing. J. Appl. Phys..

[22] Kalish A.N., Ignatyeva D.O., Belotelov V.I., Kreilkamp L.E., Akimov I.A., Achanta V.G., Bayer M., Sukhorukov A.P. (2014). Transformation of mode polarization in gyrotropic plasmonic waveguides. Las. Phys..

[23] Zheludev N.I., Kivshar Y.S. (2012). From metamaterials to metadevices. Nat. Mater..

[24] Mao R., Wang G., Cai T., Hou H., Wang D., Wu B., Zhang Q. (2020). Tunable metasurface with controllable polarizations and reflection/transmission properties. J. Phys. D Appl. Phys..

[25] Xiao S., Wang T., Liu T., Zhou C., Jiang X., Zhang J. (2020). Active metamaterials and metadevices: A review. J. Phys. D Appl. Phys..

[26] Chernov A.I., Kozhaev M.A., Ignatyeva D.O., Beginin E.N., Sadovnikov A.V., Voronov A.A., Karki D., Levy M., Belotelov V.I. (2020). All-dielectric nanophotonics enables tunable excitation of the exchange spin waves. Nano Lett..

[27] Khramova A.E., Ignatyeva D.O., Kozhaev M.A., Dagesyan S.A., Berzhansky V.N., Shaposhnikov A.N., Tomilin S.V., Belotelov V.I. (2019). Resonances of the magneto-optical intensity effect mediated by interaction of different modes in a hybrid magnetoplasmonic heterostructure with gold nanoparticles. Opt. Express.

[28] Kuzmichev A.N., Sylgacheva D.A., Kozhaev M.A., Krichevsky D.M., Shaposhnikov A.N., Berzhansky V.N., Freire-Fernández F., Qin H.J., Popova O.E., Keller N. (2020). Influence of the plasmonic nanodisk position inside a magnetic medium on the Faraday effect enhancement. Phys. Status Solidi Rapid Res. Lett..

[29] Lodewijks K., Maccaferri N., Pakizeh T., Dumas R.K., Zubritskaya I., Åkerman J., Vavassori P., Dmitriev A. (2014). Magnetoplasmonic design rules for active magneto-optics. Nano Lett..

[30] Ignatyeva D.O., Knyazev G.A., Kapralov P.O., Dietler G., Sekatskii S.K., Belotelov V.I. (2016). Magneto-optical plasmonic heterostructure with ultranarrow resonance for sensing applications. Sci. Rep..

[31] Ignatyeva D.O., Kapralov P.O., Knyazev G.A., Sekatskii S.K., Dietler G., Alam M.N., Vasiliev M., Alameh K., Belotelov V.I. (2016). High-Q surface modes in photonic crystal/iron garnet film heterostructures for sensor applications. JETP Lett..

[32] Borovkova O.V., Ignatyeva D.O., Sekatskii S.K., Karabchevsky A., Belotelov V.I. (2020). High-Q surface electromagnetic wave resonance excitation in magnetophotonic crystals for supersensitive detection of weak light absorption in the near-infrared. Phot. Res..

[33] Merzlikin A.M., Kuznetsov E.V., Baryshev A.V. (2018). Magneto-optical device based on polarization sensitivity for perspective biosensing applications. IEEE Sens. J..

[34] Konopsky V.N. (2010). Plasmon-polariton waves in nanofilms on one-dimensional photonic crystal surfaces. New J. Phys..

[35] Višňovský Š., Nývlt M., Prosser V., Lopušník R., Urban R., Ferré J., Pénissard G., Renard D., Krishnan R. (1995). Polar magneto-optics in simple ultrathin-magnetic-film structures. Phys. Rev. B.

[36] Ignatyeva D.O., Kalish A.N., Levkina G.Y., Sukhorukov A.P. (2012). Surface plasmon polaritons at gyrotropic interfaces. Phys. Rev. A.

[37] Ignatyeva D.O., Karki D., Voronov A.A., Kozhaev M.A., Krichevsky D.M., Chernov A.I., Levy M., Belotelov V.I. (2020). All-dielectric magnetic metasurface for advanced light control in dual polarizations combined with high-Q resonances. Nat. Comm..

[38] Rheims J., Köser J., Wriedt T. (1997). Refractive-index measurements in the near-IR using an Abbe refractometer. Meas. Sci. Technol..

[39] Daimon M., Masumura A. (2007). Measurement of the refractive index of distilled water from the near-infrared region to the ultraviolet region. Appl. Opt..

[40] Hongwei L., Colleen L.N., Jason H.H. (2006). Biomedical applications of plasmon resonant metal nanoparticles. Nanomedicine.

[41] Anker J.N., Hall W.P., Lyandres O., Shah N.C., Zhao J. (2009). Biosensing with plasmonic nanosensors. Nanosci. Technol..

[42] Silvia R., Gianluigi Z., Stefania T., Giuseppe C., Erika P., Giuseppe C., Stefano C., Ivo R., Vito M. (2018). Label-free sensing of ultralow-weight molecules with all-dielectric metasurfaces supportingbound states in the continuum. Phot. Res..

[43] Noemi B., Sípová-Jungová H., Nils Länk O., Ruggero V. (2019). Plasmonic versus all-dielectric nanoantennas for refractometric sensing: A direct comparison. ACS Photonics.

